# Anemia Is an Indicator for Worse Organ Damage Trajectories in Patients with Systemic Sclerosis: A Retrospective Study

**DOI:** 10.3390/jcm11175013

**Published:** 2022-08-26

**Authors:** Zhaohua Li, Dan Xu, Xintong Jiang, Ting Li, Yin Su, Rong Mu

**Affiliations:** 1 Department of Rheumatology and Immunology, Peking University Third Hospital, No.49 Huayuan North Road, Haidian District, Beijing 100191, China; 2 Department of Rheumatology and Immunology, Peking University People’s Hospital, No.11 Xizhimen South Street, Xicheng District, Beijing 100044, China

**Keywords:** systemic sclerosis, anemia, organ damage, damage index

## Abstract

It is important for clinicians to determine the risk of worsening trajectories in SSc patients. The Scleroderma Clinical Trials Consortium (SCTC) Damage Index (DI) has been developed to quantify organ damage and shows good capability for mortality and morbidity prediction in patients with SSc. This retrospective study aimed to describe the SCTC-DI in Chinese SSc patients and to find features predicting worse organ damage trajectories based on SCTC-DI. A total of 433 SSc patients who met the inclusion criteria in the Peking University Third Hospital (PKUTH-SSc) and People’s Hospital SSc cohort (PKUPH-SSc) were recruited for our study. Organ damage was relatively mild in our Chinese SSc cohort compared to other cohorts, with a mean SCTC-DI of 5.21 ± 4.60. We used both SCTC-DI ≥ 6 and ≥4 to define the high burden of organ damage and established two risk models by the LASSO algorithm, which revealed good identification of high organ damage burden (AUC = 0.689, 95% CI 0.636 to 0.742, *p* < 0.001 in SCTC-DI ≥ 6 model; AUC = 0.694, 95% CI 0.641 to 0.746, *p* < 0.001 in modified SCTC-DI ≥ 4 model). The anemia index at the baseline was included in these two models and was also independently related to organ damage progression (HR = 1.75, 95% CI 1.16 to 2.66, *p* = 0.008). In addition, the presence of an anti-Scl-70 autoantibody was also a predictor of progression (HR = 1.91, 95% CI 1.22 to 2.99, *p* = 0.005). In conclusion, anemia at the baseline was an important indicator for worse organ damage trajectories in SSc patients. We recommend using hemoglobin as a potential biomarker to evaluate organ damage in SSc patients.

## 1. Introduction

Systemic sclerosis (SSc) is an autoimmune disease characterized by collagen deposition in different tissues, resulting in multiple organ damage and functional failure. Organ damage, defined as the permanent and irreversible loss of the anatomical structure or physiological function caused by SSc and not secondary to its treatment or comorbidities, is the main culprit of SSc-related death in multinational cohorts [1,2,3,4,5,6], including scleroderma renal crisis (SRC) [4], lung fibrosis [7,8], and pulmonary arterial hypertension (PAH) [5], and the SSc-related mortality has steadily declined, attributed to carefully assessed organ damage and the early management of SSc over the years [9,10]. Early screening for major organ involvement in SSc patients is essential and has now been recommended in clinical practice.

SSc is a heterogeneous disease characterized not only by limited and diffuse skin lesions, but also by a diverse spectrum of organ involvement, leading to significant differences in organ damage pattern, severity, progression, mortality, and treatment choices between patients [11,12,13]. Therefore, it is essential to determine the risk factors of worsening organ damage trajectories in patients with SSc. Disease activity has been reported as an independent risk factor for the severity of accrual organ damage to respiratory, cardiac, skin, and vascular systems in the EUSTAR cohort [14], and respiratory and cardiac systems in a Canadian cohort at follow-up [15]. In a Greek cohort, patients with dcSSc, DU, and esophageal involvement are more likely to develop pulmonary fibrosis at 6 years of follow-up [16]. Nevertheless, a comprehensive outcome measure is required to characterize the overall worse organ damage trajectories of SSc-related systems.

Recently, organ damage burden classified according to the Scleroderma Clinical Trials Consortium Damage Index (SCTC-DI) has been suggested in clinical studies. SCTC-DI consists of the major SSc-related organ damage of six systems [17], and each item in SCTC-DI is strongly associated with the morbidity and mortality of SSc [17]. The good discriminatory capability of SCTC-DI for both mortality risk and also morbidity has been validated in the Scleroderma Cohort Study (ASCS) and Canadian Scleroderma Research Group (CSRG) cohorts [17,18]. Very early SSc patients in ASCS and CSRG cohorts who were older, male, dcSSc, with elevated CRP and negative anti-centromere autoantibody were more likely to present with higher SCTC-DI [18]. Thus, SCTC-DI provides physicians with a multifaceted outcome measure in interventional trials and observational studies and a quantitative screening tool for patients at risk of poor prognosis in SSc [17,18,19]. However, the SCTC-DI has not yet been validated in Chinese SSc patients, and the clinical features at the baseline, which predicted worse organ damage trajectories based on the changes of SCTC-DI, have not been clarified.

In this study, we investigated the incidence and progression of major organ complications in two Chinese retrospective cohorts of SSc patients based on SCTC-DI. We reported here, for the first time, that anemia at the baseline was an indicator to discriminate against the high burden of organ damage and also predict damage progression, suggesting anemia can be used as a warning sign of the worse organ damage trajectories in SSc patients.

## 2. Materials and Methods

### 2.1. Study Population: PKUTH-SSc and PKUPH-SSc Cohort

We retrospectively identified two cohorts of patients who were diagnosed as SSc at the Department of Rheumatology and Immunology of Peking University Third Hospital (PKUTH) and Peking University People’s Hospital (PKUPH) from January 2001 to July 2021, as reported previously [20]. The Ethics Committee approved the study of Peking University Third Hospital ((2022) 142-01)) and Peking University People’s Hospital (2019PHB276-01). The informed consent from patients was waived due to the retrospective nature of the study and the anonymization of all data.

Patients from both cohorts who met the following criteria were included in the present study: (1) age > 18 years old at diagnosis; (2) fulfilled the European League Against Rheumatism (EULAR) 2013 classification criteria for SSc [21]; (3) completed history, physical exam, and available clinical and laboratory data to assess SCTC-DI accurately. Patients with at least 6 months of follow-up were enrolled for the follow-up study. The study design is presented in Figure S1.

### 2.2. Data Collection and Variables Definition

All available clinical and laboratory data were collected from electronic medical records at baseline and the follow-up time point.

Cutaneous subsets were defined as limited cutaneous SSc (lcSSc), diffuse cutaneous SSc (dcSSc), and sine scleroderma based on the criteria of LeRoy et al. [22]. SSc with overlap syndrome with rheumatoid arthritis (RA), systemic lupus erythematosus (SLE), or myositis were recorded as overlap syndrome. Disease duration was defined as the time since the first SSc-related manifestation onset (including Raynaud’s phenomenon (RP) symptoms or non-RP symptoms related to SSc) [23]. Treatments including corticosteroids and immunosuppressants were recorded.

Furthermore, autoantibody profiles were collected, including anti-nuclear (ANA), anti-topoisomerase 1 (Scl-70), and anti-centromere proteins (CENP A and B). Serum inflammatory parameters including C-reactive protein (CRP), erythrocyte sedimentation rate (ESR), complement 3 (C3), and C4 were collected. The standard Westergren method was used to adjust ESR for hematocrit according to the Minimal Validation Procedures and Performance Criteria for manufacturers of alternate ESR methods, based on the recommendation of the International Council for Standardization in Hematology (ICSH) [24]. CRP elevation was defined as CRP > 5mg/L, and ESR elevation was defined as ESR > 20 mm/h. Hypocomplementemia was defined as C3 < 0.8 g/L and/or C4 < 0.1 g/L. The diagnosis of anemia was based on the reduced hemoglobin (Hb) concentrations (<120 g/L for females and <130 g/L for males) [25].

### 2.3. Outcome Definition

SCTC-DI was calculated as previously reported [17]. We used the criterion, “any peak tricuspid regurgitation velocity > 3.4m/s measured by echocardiogram” [26], as a surrogate criterion for pulmonary arterial hypertension (PAH) if right heart catheterization had not been carried out.

Patients were divided into the low and high burden of organ damage groups based on the SCTC-DI score at baseline. The cut-off value of 6 was determined by the original classification of the SCTC risk group [17]. SCTC-DI score ≥ 6, which implies a moderate to high mortality risk, was identified as a high burden of organ damage, while an SCTC-DI score < 6 was defined as a low burden. Given the different distribution of SCTC-DI between our cohort and the Canadian and Australian cohort, we also employed the cut-off value of organ damage burden based on the median of SCTC-DI in our cohort, determining SCTC-DI score ≥ 4 as the high burden group.

For the follow-up analysis, ΔSCTC-DI ≥ 1 was defined as organ damage progression [19]. Besides the overall progression, organ damage progression was also separated into organ systems, including the progression of musculoskeletal and skin, vascular, gastrointestinal, respiratory, cardiovascular, and renal systems.

### 2.4. Statistical Analysis

LASSO algorithm was performed to select and sort the statistically significant clinical features [27]. All unordered categorical variables were identified as dummy variables. Ten-fold cross-validation on the training set to calculate the weight of the LASSO penalty (denoted as lambda). The lambda of the minimum partial likelihood deviance and one standard error of the cross-validated errors for lambda-min was used for feature selection. R studio and the “glmnet” package were used to perform the LASSO logistic regression and Cox survival analysis to develop the evaluation model of organ damage burden and the prediction model for organ damage progression based on SCTC-DI. The model’s discrimination and calibration were internally evaluated based on the area under the curve (AUC) of the receiver operating characteristic and calibration plots.

We compared the baseline characteristics between low and high burden groups and with and without anemia groups using the student *t*-test between continuous variables, while the Chi-Square test and Fisher’s exact test were used for categorical variables. We compared the frequency of SCTC-DI items between our cohorts, ASCS, and CSRG cohorts using the Chi-Square or Fisher’s exact test. Statistical analysis was performed using SPSS software (version 26.0, IBM Corp., Chicago, IL, USA), R Studio Server (R Studio, PBC., version 4.1.3, Boston, MA, USA), and GraphPad Prism software (version 9.0.0, GraphPad Software LLC., San Diego, CA, USA), and a *p*-value < 0.05 was considered statistically significant.

## 3. Results

### 3.1. Baseline Characteristics of PKUTH-SSc and PKUPH-SSc Cohort

A total of 433 SSc patients fulfilling the selection criteria were enrolled in our study from PKUTH-SSc and PKUPH-SSc cohorts (Figure S1). The baseline characteristics of the study cohorts were shown in Table 1. Of these, 373 (86.1%) were female, with an average age of 52.0 ± 14.4 years at baseline. The duration since symptom onset was 8.2 ± 9.5 years. A total of 40.9% of dcSSc, 40.0% of lcSSc, and 16.2% of SSc with overlap syndrome patients were recruited in our cohort.

Then, we compared our cohort with ASCS and CSRG cohorts and found that our cohort had similar gender proportions (86.7% female in the ASCS cohort and 86.4% in the CSRG cohort), but was older (44.0 ± 15.3 years in the ASCS cohort and 45.9 ± 13.7 years in the CSRG cohort). The disease duration of our cohort was similar to the CSRG cohort (9.6 ± 9.3 years), but shorter than the ASCS cohort (13.1 ± 12.7 years). The proportion of dcSSc patients was higher in our cohort (24.5% in the ACSC cohort and 36.7% in the CSRG cohort) [17].

SCTC-DI was quantified as described in Method, and the incident frequency of each SCTC-DI item was shown in Table 2. The SCTC-DI score was 5.21 ± 4.60 in our cohort, with a median of 4.00. Among all items, ILD > 20% extent on HRCT (39.7%), sicca symptoms (37.0%), and underweight or weight loss (35.8%) were the top three most common items of SCTC-DI in our study population.

Moreover, the SCTC-DI score was lower in our cohort compared with the ASCS (mean 6.65 ± 4.80, and median 6.00) and CSRG cohorts (mean 6.90 ± 4.70, and median 6.00) [17]. Compared with the ASCS and CSRG cohorts (Table S1) [17], organ damage in our cohort was milder except for the respiratory system. Although the gastrointestinal system was the most frequently affected in all these three cohorts, the incidence of gastroesophageal reflux disease in our cohort (23.8%) was only half of that in the ASCS (47.6%) and CSRG (63.4%) cohorts, consistent with the different incidence of musculoskeletal and skin system damage. In contrast, the incidence of moderate to severe ILD was higher in our cohort (39.7%) than that in the ASCS (9.0%) and CSRG (22.5%) cohorts.

### 3.2. Anemia as an Indicator of High-Burden Organ Damage

When the high burden is categorized as SCTC-DI ≥ 6, the elderly (*p* < 0.001), longer disease duration (*p* = 0.003), overlap syndrome with DM/PM (*p* = 0.007), anemia (*p* < 0.001), ESR elevation (*p* = 0.003), and the usage of steroids (*p* = 0.013) were more often in the high-burden group compared to the low-burden group at the univariable analysis (Table S2). Other clinical characteristics had no significant difference between the high-burden and low-burden groups.

Referring to the Canadian and Australian cohort study [17], we also chose the median SCTC-DI score in our cohort as the cut-off value for the organ damage burden modified classification (SCTC-DI ≥ 4 as the high-burden group). Other than the factors mentioned above, females (*p* = 0.028) and patients overlapping with RA (*p* = 0.039) were more likely to have a higher burden of organ damage based on the modified classification (as shown in Table S3).

**Figure 1:**
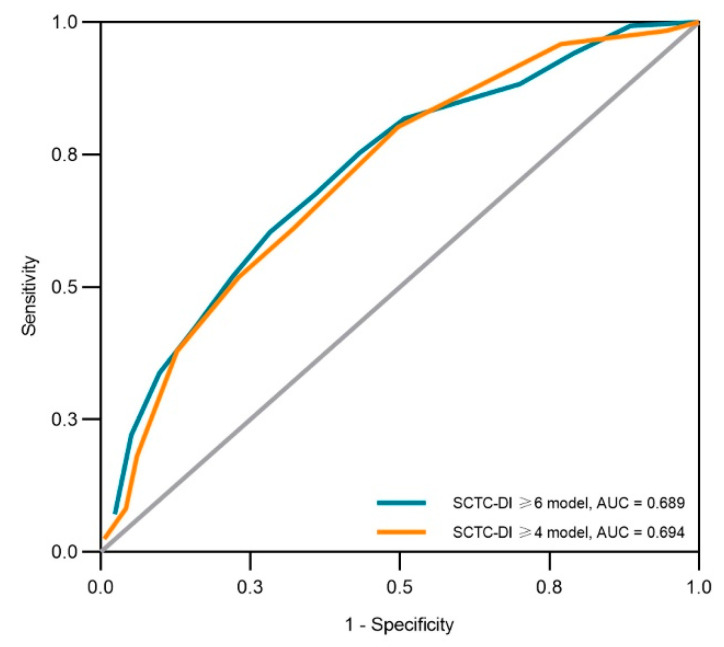
Evaluation of the regression model for high organ damage burden. Receiver operating characteristic (ROC) curves for the multivariate logistic model discriminating Scleroderma Clinical Trials Consortium Damage Index (SCTC-DI) low- and high-burden groups using the original beta weights of variables. Anemia, age, and steroid usage were included in the SCTC-DI ≥ 6 model. Anemia and disease duration were included in the SCTC-DI ≥ 4 model. AUC, area under the curve.

The LASSO logistic regression was used, as outlined in Method, to discover the independent risk factors associated with high organ damage burden in SSc patients. Variables that showed significant differences between high and low burden groups were enrolled in the LASSO regression to identify the most critical risk factors. Patients missing data on ESR were excluded from the regression. As shown in Table 3, anemia (OR = 2.30, 95% CI 1.70 to 4.00, *p* < 0.001 in SCTC-DI ≥ 6 model; OR = 1.89, 95% CI 1.24 to 2.90, *p* = 0.003 in SCTC-DI ≥ 4 modified model) was the only independent risk factor associated with the high burden organ damage according to both the original and modified classifications. Age and steroids usage at the baseline were selected in the final evaluating model of the original high organ damage burden, while disease duration was selected for the modified high organ damage burden. Their associated coefficients in the final logistic model are illustrated in Table 2. There were no interactions among the variables in the model, and the variance inflation factor (VIF) of all features in the model was near one. The ROC analysis revealed a good discriminatory capability of assessing the organ damage burden with an area under the curve (AUC) of 0.689 (95% CI 0.636 to 0.742, *p* < 0.001) in the original high-burden model and 0.694 (95% CI 0.641 to 0.746, *p* < 0.001) in the modified high-burden model using the original beta weights of these variables (Figure 1). Taken together, anemia was an indicator of a high organ damage burden in both the original and modified classifications. Age, disease duration, and steroid usage at the baseline were also associated with a high organ damage burden.

### 3.3. Anemia at the Initial Visit as a Risk Factor for Organ Damage Progression

To verify the progression of organ damage during follow-up based on SCTC-DI, 207 patients with at least 6 months of follow-up data were enrolled for this analysis. The mean follow-up time was 2.04 ± 1.56 years. One hundred (48.3%) patients went through organ damage progression (ΔSCTC-DI ≥ 1) during follow-up (Table S4). Gastrointestinal (19.8%) and respiratory (18.8%) systems were the two most common organ systems with accrual damage in our cohort. Moderate to severe ILD (15.9%) was the most frequently new-onset manifestation.

**Figure 2:**
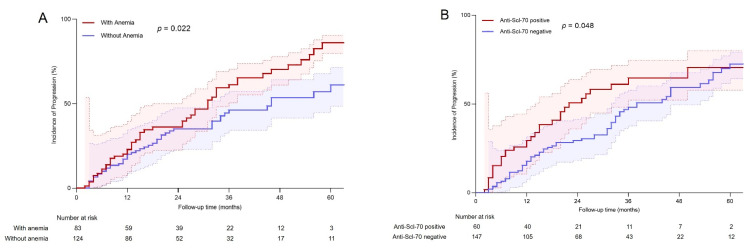
Univariate Cox cumulative incidence plot for time to organ damage progression in SSc patients. (**A**) With or without anemia; (**B**) positive or negative anti-Scl-70 autoantibody. SSc, systemic sclerosis.

**Figure 3:**
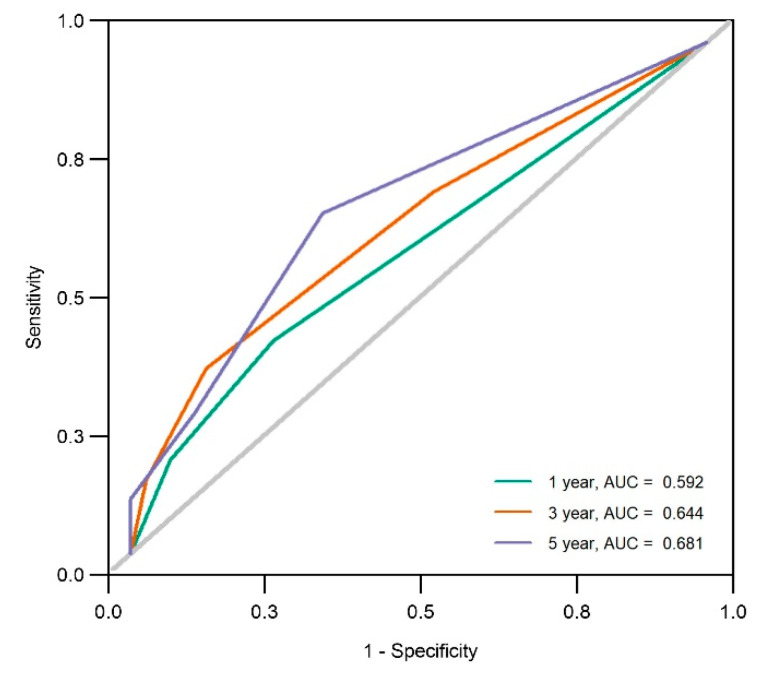
Time-dependent ROC curves for the final prediction model of organ damage progression. The final prediction model included anemia at the initial visit and positive anti-Scl-70 autoantibody. AUC, area under the curve.

In univariate Cox analyses, we identified that four baseline features were significantly associated with organ damage progression (Table S5), including anemia at the baseline (HR = 1.59, 95% CI 1.07 to 2.36, *p* = 0.022), ESR elevation (HR = 1.54, 95% CI 1.03 to 2.31, *p* = 0.036), CRP elevation (HR = 1.74, 95% CI 1.12 to 2.69, *p* = 0.014), and the presence of an anti-Scl-70 autoantibody (HR = 1.53, 95% CI 1.00 to 2.34, *p* = 0.048). Finally, anemia at the baseline (HR = 1.75, 95% CI 1.16 to 2.66, *p* = 0.008) and the anti-Scl-70 autoantibody (HR = 1.91, 95% CI 1.22 to 2.99, *p* = 0.005) were independently related to organ damage progression. The univariate Cox cumulative incidence plots were shown in Figure 2. The final prediction model included anemia, and the anti-Scl-70 autoantibody showed a good predictive value. The AUC for the five-year progression incidence was 0.681 (95% CI 0.513 to 0.848, *p* < 0.001) (Figure 3). Thus, anemia at the baseline and the anti-Scl-70 autoantibody were key predictors for organ damage progression in patients with SSc.

### 3.4. Anemia Is Associated with the Inflammation of SSc

Given that anemia could indicate a high organ damage burden and a predictor of five-year organ damage progression, we further explored if SSc patients with anemia at the baseline had distinct clinical features. As shown in Table 4, SSc patients with anemia had higher ESR (56.6% in anemia vs. 25.5% in non-anemia, *p* < 0.001) and CRP levels (28.1% in anemia vs. 17.2% in non-anemia, *p* = 0.010). Hypocomplementemia (52.5% in anemia vs. 39.7% in non-anemia, *p* = 0.013) was also more common in patients with anemia, indicating that anemia might be associated with disease activity in SSc.

For the organ damage characteristics, SSc patients with anemia were more likely to develop damage of the musculoskeletal and skin (60.6% in anemia group vs. 39.5% in non-anemia group, *p* < 0.001), gastrointestinal (62.3% vs. 44.6%, *p* < 0.001), cardiovascular (16.6% vs. 10.1%, *p* = 0.046), and renal system (3.4% vs. 0.0%, *p* = 0.004) compared to those without anemia (Table S6). During follow-up, the organ damage progression of the cardiovascular system (12.0% in anemia vs. 3.2% in non-anemia, *p* = 0.013) and the renal system (3.6% vs. 0.0%, *p* = 0.033) were more common in patients with anemia (Table S7). To summarize, anemia was strongly associated with worse organ damage trajectories in SSc patients.

### 3.5. Anemia-Related Worse Organ Damage Trajectories within the SSc Subtypes

Among all three subsets, including lcSSc, dcSSc, and SSc with overlap syndrome, patients with anemia had a significantly higher organ damage burden and worse progression. However, 23.4% of patients with anemia were SSc with overlap syndrome. We wondered if anemia was still an essential indicator of worse organ damage trajectories in SSc after removing patients with overlap syndrome.

In terms of the burden of organ damage assessment, the ROC analysis revealed a similar good discriminatory capability with an AUC of 0.696 (95% CI 0.638 to 0.754, *p* < 0.001) in the original high-burden (≥6) model and 0.721 (95% CI 0.666 to 0.776, *p* < 0.001) in the modified high-burden (≥4) model using the modified beta weights of variables, showed in Table 3. These results supported the cueing role of baseline anemia in evaluating organ damage severity.

In terms of organ damage progression, the anti-Scl-70 autoantibody (HR = 2.02, 95% CI 1.13 to 3.62, *p* = 0.002) was also independently related to organ damage progression, while anemia at the baseline (HR = 1.30, 95% CI 0.78 to 2.14, *p* = 0.479) was not associated with organ damage progression. Given the strong correlation between anemia and baseline SCTC-DI, we sub-grouped according to the baseline severity of SCTC-DI and found that anemia was an independent risk factor for the progression of organ damage in the high-burden SCTC-DI group (HR = 3.46, 95% CI 1.23 to 9.72, *p* = 0.019 in SCTC-DI ≥ 6 group; HR = 2.32, 95% CI 1.16 to 4.65, *p* = 0.017 in SCTC-DI ≥ 4 group). In contrast, in patients with overlap syndrome, anemia was independently related to the worse trajectories in the low-burden SCTC-DI group (HR = 3.06, 95% CI 1.14 to 8.24, *p* = 0.027 in SCTC-DI < 6 group; HR = 5.93, 95% CI 1.24 to 28.48, *p* = 0.026 in SCTC-DI < 4 group). Taken together, our results showed that patients with baseline anemia might represent a subgroup at risk of worse organ damage trajectories in SSc with or without the overlap syndrome subset.

## 4. Discussion

This is the first study to describe the SCTC-DI characteristics of Chinese patients with SSc. Compared to the western cohorts, the organ damage burden is lower in Chinese SSc patients, except for the respiratory system. Interestingly, we found that anemia at the baseline was the key potential indicator of a worse organ damage trajectory. Moreover, anemia was also associated with the inflammatory status in SSc.

In our study, we found a lower incidence of organ damage in the majority of the involved systems, especially the musculoskeletal and skin, vascular, and gastrointestinal systems, but a higher prevalence of SSc-ILD in Chinese SSc patients. Different genetic backgrounds may result in the heterogeneity of organ damage in SSc patients. A similar result was found in a retrospective study of a multiethnic SSc cohort in Toronto, which showed patients of Chinese descent had milder organ damage than European-descent patients, including less frequent joint and gastrointestinal involvements, less severe digital ulcers, PAH, and the absence of renal involvement [28]. Furthermore, East Asian patients also less frequently have calcinosis and esophageal dysmotility in a longitudinal Canadian SSc cohort compared with other descent patients [29]. In contrast, SSc-ILD is more common in Asian patients than in patients of other ethnicities [30]. SSc-ILD was also more common in Chinese [5] or Asian [31] patients than European patients in cross-sectional research across racial groups in the EUSTAR cohort, while SSc-ILD is less prevalent and milder in Caucasians [30,32]. The incidence of moderate-to-severe ILD was as low as 9.0% in the ASCS cohort, which may be related to the higher proportion of Caucasians in the ASCS cohort (94.1%) than in the CSRG cohort (90.2%) [33]. All of this evidence suggests that organ involvements have racial differences. Different demographic and clinical characteristics of the cohorts may also lead to the heterogeneity of organ damage. In comparing the baseline characteristics of our cohort with these two cohorts, we observe that our cohort included more elderly and dcSSc patients, which may also result in a high incidence of ILD [34]. Moreover, consistent with the lower incidence of ILD in the ASCS cohort, fewer patients in the ASCS cohort had dcSSc. Thus, in the strategy of treatment and the design of clinical trials in multinational research, differences in organ damage patterns among populations should be taken into consideration.

In the further relevant variables analysis for high organ damage burden, we found that anemia was the only independent risk factor consistently validated in different models, no matter whether we defined high burden as SCTC-DI ≥ 6 or ≥4, which was the median SCTC-DI score in our cohort. Interestingly, we found that anemia was not only an indicator of high organ damage burden, but also a predictor of organ damage progression. It was initially reported in 1991 that SSc patients with lower hemoglobin levels were at risk of a shortened survival [35]. Several researchers have reported that the hemoglobin level was an independent risk factor for 3-to-5-year mortality in early dcSSc patients [36], in-hospital mortality [37], severe skin thickness [38], as well as higher healthcare costs related to organ damage [39] in SSc patients. In addition, patients with anemia also showed a more significant progression of organ damage during follow-up, which has been reported in our previous cohort study [20], and shows that anemia was a predictor for the renal involvement of SSc. In our results, baseline anemia was related to the progression of organ damage in the high-burden SCTC-DI group among lcSSc and dcSSc patients and in the low-burden SCTC-DI group of SSc patients with overlap syndrome. This may be relevant to the heterogeneity of disease progression between subgroups [40]. Unfortunately, anemia was not included in the risk factor analysis of organ damage in other cohort studies based on SCTC-DI [18], indicating that this widely available and inexpensive test has been overlooked in clinical practice.

There are several possible mechanisms for the correlation between anemia and organ damage. On the one hand, anemia results from organ damage, such as microangiopathic hemolysis related to the renal crisis [41], absolute iron deficiency anemia related to gastrointestinal disorders [42], etc. On the other hand, the dysregulated hematopoietic process is associated with organ damage in fibrotic diseases. Firstly, erythropoietin (Epo)-producing fibroblasts in the kidney transdifferentiate into myofibroblasts during kidney fibrosis, leading to anemia due to Epo deficiency [43,44]. Secondly, altered fibrosis-related cytokines, such as interleukin-6 (IL-6) and transforming growth factor-β (TGF-β), contribute to the deficiency and dysfunction of hematopoietic stem cells (HSCs) that ultimately result in decreased erythropoiesis [45]. Thirdly, in our study, anemia was strongly associated with the inflammatory index. Under the chronic inflammation condition, the production and/or biological activity of the hormone erythropoietin were reduced, and the iron distribution was dysregulated because of the abnormal macrophage activation [46]. Furthermore, the enhanced chronic Toll-like receptor 9 signaling in SSc may also contribute to anemia via the differentiation of dysfunctional hemophagocytes [47,48]. Since SSc patients with persistent inflammation were characterized by more severe respiratory and cardiovascular manifestations [49], anemia of inflammation is closely related to organ damage. Taken together, anemia may be not only the result, but also the indicator of organ damage in SSc. We recommend using hemoglobin as a potential biomarker for determining worse organ damage trajectories in SSc patients.

In addition, SSc-related autoantibodies are also associated with organ manifestations. Patients with anti-Scl-70 autoantibodies had progressive organ damage during follow-up in our cohort, while early SSc patients with ACA had better damage trajectories in the ASCS and CSRG cohorts [18]. The anti-RNA-polymerase-III (anti-RNAP III) antibody is also one of the SSc-related autoantibodies with high specificity and is strongly related to renal involvement [50,51]. However, our study did not include anti-RNAP III antibodies due to their low prevalence (only 5.93%) in Chinese SSc patients [52] and the lack of large-scale clinical testing for anti-RNAP III antibodies in Chinese hospitals.

Our study has several limitations. Firstly, based on the trait of a retrospective study, incomplete clinical and laboratory information is common in our cohort. For example, detailed data for the cause of anemia were not conducted, such as serum transferrin, ferritin, hepcidin, Coombs’ test, bone marrow test, etc. A more comprehensive anemia diagnosis should be included in further investigations to determine the relationship between anemia of inflammation and organ damage. Secondly, treatments and responsiveness to treatment are also important prognostic factors. As we only included the usage of steroids and immunosuppressive agents as independent parameters, their role in the progression of different organ damages has not been fully demonstrated. Further research regarding the treatment response is warranted. Nevertheless, through the multi-center retrospective cohort and follow-up data, our study provided a reference for further research of systemic organ damage based on SCTC-DI.

## 5. Conclusions

This study, based on the PKUTH-SSc and PKUPH-SSc cohorts, is the first retrospective study describing the characteristics of SCTC-DI in Chinese SSc patients. We found that the organ damage was mild in our cohort, and the pattern of involved organs was slightly different compared to other cohorts. Moreover, our results highlight the value of anemia at the baseline in identifying patients at high risk of worse organ damage trajectories in SSc, suggesting that the monitoring of hemoglobin might help predict the prognosis in patients with SSc.

## Figures and Tables

**Table 1:** Frequency of SCTC-DI items in SSc patients.

Characteristics	Baseline (N = 433)	Follow-Up (N = 207)	*p* Value
Age at initial visit (years)	52.0 ± 14.4	51.0 ± 15.1	0.389
Sex, female	373 (86.1%)	180 (87.0%)	0.779
Disease duration (years)	8.2 ± 9.5	8.6 ± 9.8	0.59
Disease classification			
lcSSc	173 (40.0%)	67 (32.4%)	0.064
dcSSc	177 (40.9%)	93 (44.9%)	0.332
Sine scleroderma	13 (3.0%)	3 (1.4%)	0.239
Overlap syndrome	70 (16.2%)	44 (21.3%)	0.115
RA	27 (6.2%)	18 (8.7%)	0.255
SLE	29 (6.7%)	18 (8.7%)	0.365
DM/PM	14 (3.2%)	8 (3.9%)	0.682
Laboratory parameters			
Anemia	175 (40.4%)	83 (40.1%)	0.939
High ESR	156/409 (38.1%)	78/192 (40.6%)	0.56
High CRP	85/393 (21.6%)	46/190 (24.2%)	0.484
Hypocomplementemia	176/391 (45.0%)	77/190 (40.5%)	0.306
Autoantibody profile			
ANA	312 (72.1%)	152 (73.4%)	0.716
Anti-topoisomerase 1	129 (29.8%)	60 (29.0%)	0.834
(Anti-Scl-70)			
Anti-centromere proteins	56 (12.9%)	31 (15.0%)	0.481
Medication			
Steroids	191 (44.1%)	77 (37.2%)	0.097
Immunosuppressants	165 (38.1%)	73 (35.5%)	0.487

N = 433 in baseline population, N = 207 in follow-up population if not specified. Abbreviations: SSc, systemic sclerosis; lcSSc, limited cutaneous systemic sclerosis; dcSSc, diffuse cutaneous systemic sclerosis; RA, rheumatoid arthritis; SLE, systemic lupus erythematous; DM, dermatomyositis; PM, polymyositis; ESR, erythrocyte sedimentation rate; CRP: C-reactive protein; ANA, anti-nuclear antibody.

**Table 2:** Frequency of SCTC-DI items in SSc patients.

Items	Baseline Assessment N = 433
Musculoskeletal and skin	208 (48.0%), Score: 1.69 ± 2.03
Joint contracture (small joints)	56 (12.9%)
Joint contracture (large joints)	7 (1.6%)
Sicca symptoms	160 (37.0%)
Proximal muscle weakness	32 (7.4%)
Calcinosis complicated by infection or requiring surgery	7 (1.6%)
Vascular	87 (20.1%), Score: 0.45 ± 0.91
Digital ulceration	87 (20.1%)
Digital amputation required	19 (4.4%)
Gastrointestinal	224 (51.7%), Score: 1.16 ± 1.35
Esophageal dysmotility	69 (15.9%)
Esophageal stricture	2 (0.5%)
Refractory gastro-esophageal reflux disease (heartburn)	103 (23.8%)
GAVE	0 (0.0%)
Pseudo-obstruction	6 (1.4%)
BMI < 18.5 kg/m^2^ or weight loss > 10% in the last 12 months	155 (35.8%)
Respiratory	176 (40.6%), Score: 1.38 ± 2.21
ILD > 20% extent on HRCT	172 (39.7%)
FVC < 70%	52 (12.0%)
Dependence on home oxygen	9 (2.1%)
Cardiovascular	55 (12.7%), Score: 0.49 ± 1.70
PAH	42 (9.7%)
Moderate to severe right ventricular dysfunction	10 (2.3%)
Myocardial disease	22 (5.1%)
Moderate to large pericardial effusion	13 (3.0%)
Renal	5 (1.2%), Score: 0.05 ± 0.41
History of SRC	5 (1.2%)
eGFR < 45mL/min/1.73m^2^	4 (0.9%)
CKD stage 5 and need for renal replacement therapy	1 (0.2%)
SCTC-DI	5.21 ± 4.60
SCTC-DI = 0 (Baseline)	68 (15.7%)

Abbreviations: SCTC-DI, Scleroderma Clinical Trials Consortium Damage Index; SSc: systemic sclerosis; GAVE, Gastric antral vascular ectasia; BMI, body mass index; ILD, interstitial lung disease; HRCT, HRCT, high-resolution CT; FVC, Forced Vital Capacity; PAH, pulmonary arterial hypertension; SRC, scleroderma renal crisis; eGFR, Estimated Glomerular Filtration Rate; CKD, chronic kidney disease.

**Table 3:** Final logistic regression model to identify high-burden organ damage in SSc.

Characteristics at Baseline	High Burden: SCTC-DI ≥ 6				Modified High Burden: SCTC-DI ≥4			
	*β*	SE	OR (95% CI)	*p* Value	*β*	SE	OR (95% CI)	*p* Value
Age (years)	0.03	0.01	1.04 (1.02, 1.05)	<0.001	-	-	-	-
Disease Duration (years)	-	-	-	-	0.06	0.01	1.06 (1.03, 1.09)	<0.001
Steroids usage	0.66	0.22	1.93 (1.25, 2.99)	0.003	-	-	-	-
Anemia	0.95	0.22	2.60 (1.70, 4.00)	<0.001	0.64	0.22	1.89 (1.24, 2.90)	0.003
Constant	−3.04	0.49	0.05 (0.02, 0.12)	<0.001	−0.29	0.16	0.75 (0.55, 1.03)	0.073

Abbreviations: SSc, systemic sclerosis; SCTC-DI, Scleroderma Clinical Trials Consortium Damage Index; *β*, the regression coefficient of a logistic regression model; SE, standard error; OR: odds ratio.

**Table 4:** Clinical characteristics of SSc patients with and without anemia.

Characteristics	Anemia	Non-Anemia	*p* Value
	N = 175	N = 258	
Age at initial visit (years)	53.03 ± 15.93	51.36 ± 13.28	0.253
Sex, female	156 (89.1%)	217 (84.1%)	0.137
Disease duration (years)	9.15 ± 9.80	7.55 ± 9.25	0.085
Disease classification			
lcSSc	69 (39.4%)	104 (40.3%)	0.854
dcSSc	60 (34.3%)	117 (45.3%)	0.022
Sine scleroderma	5 (2.9%)	8 (3.1%)	0.884
Overlap syndrome	41 (23.4%)	29 (11.2%)	0.001
RA	18 (10.3%)	9 (3.5%)	0.004
SLE	15 (8.6%)	14 (5.4%)	0.199
DM/PM	8 (4.6%)	6 (2.3%)	0.195
Inflammatory index			
High ESR	94/166 (56.6%)	62/243 (25.5%)	<0.001
High CRP	45/160 (28.1%)	40/233 (17.2%)	0.01
Hypocomplementemia	85/162 (52.5%)	91/229 (39.7%)	0.013
Autoantibody profile			
ANA	133 (76.0%)	179 (69.4%)	0.132
Anti-topoisomerase 1 (Anti-Scl-70)	52 (29.7%)	77 (29.8%)	0.977
Anti-centromere proteins	19 (10.9%)	37 (14.3%)	0.289
Medication			
Steroids	76 (43.4%)	115 (44.6%)	0.814
Immunosuppressants	64 (36.6%)	101 (39.1%)	0.588
SCTC-DI (Baseline)	6.49 ± 5.12	4.34 ± 4.00	<0.001

N = 175 in anemia group and N = 258 in non-anemia group if not specified. Abbreviations: SSc, systemic sclerosis; lcSSc, limited cutaneous systemic sclerosis; dcSSc, diffuse cutaneous systemic sclerosis; RA, rheumatoid arthritis; SLE, systemic lupus erythematosus; DM, dermatomyositis; PM, polymyositis; ESR, erythrocyte sedimentation rate; CRP: C-reactive protein; ANA, antinuclear antibody; SCTC-DI, Scleroderma Clinical Trials Consortium Damage Index.

## Data Availability

Not applicable.

## Supplementary Materials

The following supporting information can be downloaded at: https://www.mdpi.com/article/10.3390/jcm11175013/s1, Table S1: Frequency of SCTC-DI items in different SSc cohorts; Table S2: Clinical characteristics at baseline of SCTC-DI low-burden (<6) and high-burden (≥6) group; Table S3: Clinical characteristics of SCTC-DI modified low-burden (<4) and high-burden (≥4) group; Table S4: Frequency of SCTC-DI items in SSc patients during follow-up (N = 207); Table S5: Univariate Cox regression analysis of the organ damage progression based on SCTC-DI (N = 207); Table S6: Organ damage incident in different organ systems in SSc patients with and without anemia; Table S7: Organ damage progression incident in different organ systems in SSc patients with and without anemia; Figure S1: Study diagram. A simple study flow diagram of our study.

## References

[1] Jaafar S., Lescoat A., Huang S.Y., Gordon J., Hinchcliff M., Shah A.A., Assassi S., Domsic R., Bernstein E.J., Steen V. (2021). Clinical characteristics, visceral involvement, and mortality in at-risk or early diffuse systemic sclerosis: A longitudinal analysis of an observational prospective multicenter US cohort. Arthritis Res. Ther..

[2] Hao Y., Hudson M., Baron M., Carreira P., Stevens W., Rabusa C., Tatibouet S., Carmona L., Joven B.E., Huq M. (2017). Early Mortality in a Multinational Systemic Sclerosis Inception Cohort. Arthritis Rheumatol..

[3] Elhai M., Meune C., Boubaya M., Avouac J., Hachulla E., Balbir-Gurman A., Riemekasten G., Airò P., Joven B., Vettori S. (2017). Mapping and predicting mortality from systemic sclerosis. Ann. Rheum. Dis..

[4] Pokeerbux M.R., Giovannelli J., Dauchet L., Mouthon L., Agard C., Lega J.C., Allanore Y., Jego P., Bienvenu B., Berthier S. (2019). Survival and prognosis factors in systemic sclerosis: Data of a French multicenter cohort, systematic review, and meta-analysis of the literature. Arthritis Res. Ther..

[5] Hu S., Hou Y., Wang Q., Li M., Xu D., Zeng X. (2018). Prognostic profile of systemic sclerosis: Analysis of the clinical EUSTAR cohort in China. Arthritis Res. Ther..

[6] Czirják L., Kumánovics G., Varjú C., Nagy Z., Pákozdi A., Szekanecz Z., Szűcs G. (2008). Survival and causes of death in 366 Hungarian patients with systemic sclerosis. Ann. Rheum. Dis..

[7] Becker M., Graf N., Sauter R., Allanore Y., Curram J., Denton C.P., Khanna D., Matucci-Cerinic M., Pena J.D., Pope J.E. (2019). Predictors of disease worsening defined by progression of organ damage in diffuse systemic sclerosis: A European Scleroderma Trials and Research (EUSTAR) analysis. Ann. Rheum. Dis..

[8] Hoffmann-Vold A.M., Allanore Y., Alves M., Brunborg C., Airó P., Ananieva L.P., Czirják L., Guiducci S., Hachulla E., Li M. (2021). Progressive interstitial lung disease in patients with systemic sclerosis-associated interstitial lung disease in the EUSTAR database. Ann. Rheum. Dis..

[9] Yen E.Y., Singh D.R., Singh R.R. (2021). Trends in Systemic Sclerosis Mortality Over Forty-Eight Years, 1968-2015: A US Population-Based Study. Arthritis Care Res..

[10] Ratanawatkul P., Solomon J.J., Kim D., George M.P., Matarrese McGibbon L.R., Demoruelle M.K., Maleki-Fischbach M., Amigues I., Kastsianok L., Fernandez Perez E.R. (2020). Trends in systemic sclerosis and systemic sclerosis-related pulmonary arterial hypertension mortality in the USA. ERJ Open Res..

[11] Sobanski V., Giovannelli J., Allanore Y., Riemekasten G., Airò P., Vettori S., Cozzi F., Distler O., Matucci-Cerinic M., Denton C. (2019). Phenotypes Determined by Cluster Analysis and Their Survival in the Prospective European Scleroderma Trials and Research Cohort of Patients With Systemic Sclerosis. Arthritis Rheumatol..

[12] Wang Y., Franks J.M., Yang M., Toledo D.M., Wood T.A., Hinchcliff M., Whitfield M.L. (2020). Regulator combinations identify systemic sclerosis patients with more severe disease. JCI Insight.

[13] Skaug B., Khanna D., Swindell W.R., Hinchcliff M.E., Frech T.M., Steen V.D., Hant F.N., Gordon J.K., Shah A.A., Zhu L. (2020). Global skin gene expression analysis of early diffuse cutaneous systemic sclerosis shows a prominent innate and adaptive inflammatory profile. Ann. Rheum. Dis..

[14] Fasano S., Riccardi A., Messiniti V., Caramaschi P., Rosato E., Maurer B., Smith V., Siegert E., De Langhe E., Riccieri V. (2019). Revised European Scleroderma Trials and Research Group Activity Index is the best predictor of short-term severity accrual. Ann. Rheum. Dis..

[15] Nevskaya T., Baron M., Pope J.E., Canadian Scleroderma Research Group (2017). Predictive value of European Scleroderma Group Activity Index in an early scleroderma cohort. Rheumatology.

[16] Panopoulos S., Bournia V.K., Konstantonis G., Fragiadaki K., Sfikakis P.P., Tektonidou M.G. (2018). Predictors of morbidity and mortality in early systemic sclerosis: Long-term follow-up data from a single-centre inception cohort. Autoimmun. Rev..

[17] Ferdowsi N., Huq M., Stevens W., Hudson M., Wang M., Tay T., Burchell J.L., Mancuso S., Rabusa C., Sundararajan V. (2019). Development and validation of the Scleroderma Clinical Trials Consortium Damage Index (SCTC-DI): A novel instrument to quantify organ damage in systemic sclerosis. Ann. Rheum. Dis..

[18] Barbacki A., Baron M., Wang M., Zhang Y., Stevens W., Sahhar J., Proudman S., Nikpour M., Man A. (2022). Damage Trajectories in Systemic Sclerosis Using Group-Based Trajectory Modeling. Arthritis Care Res..

[19] Zheng B., Wang M., Stevens W., Proudman S., Nikpour M., Baron M., Canadian Scleroderma Research G., Australian Scleroderma Interest G. (2022). Associations between the Composite Response Index in Diffuse Cutaneous Systemic Sclerosis (CRISS), survival and other disease measures. Semin. Arthritis Rheum..

[20] Xu D., Zhu L., Cai R., Yi Z., Zhang H., Guo G., Liu S., Xu J., Wang Q., Su Y. (2021). A multi-predictor model to predict risk of scleroderma renal crisis in systemic sclerosis: A multicentre, retrospective, cohort study. Clin. Exp. Rheumatol..

[21] van den Hoogen F., Khanna D., Fransen J., Johnson S.R., Baron M., Tyndall A., Matucci-Cerinic M., Naden R.P., Medsger T.A., Carreira P.E. (2013). 2013 classification criteria for systemic sclerosis: An American College of Rheumatology/European League against Rheumatism collaborative initiative. Arthritis Rheum..

[22] LeRoy E.C., Black C., Fleischmajer R., Jablonska S., Krieg T., Medsger T.A., Rowell N., Wollheim F. (1988). Scleroderma (systemic sclerosis): Classification, subsets and pathogenesis. J. Rheumatol..

[23] Domsic R.T., Gao S., Laffoon M., Wisniewski S., Zhang Y., Steen V., Lafyatis R., Medsger T.A. (2021). Defining the optimal disease duration of early diffuse systemic sclerosis for clinical trial design. Rheumatology.

[24] Kratz A., Plebani M., Peng M., Lee Y.K., McCafferty R., Machin S.J., International Council for Standardization in H. (2017). ICSH recommendations for modified and alternate methods measuring the erythrocyte sedimentation rate. Int. J. Lab. Hematol..

[25] Beutler E., Waalen J. (2006). The definition of anemia: What is the lower limit of normal of the blood hemoglobin concentration?. Blood.

[26] Kanwar M.K., Tedford R.J., Thenappan T., De Marco T., Park M., McLaughlin V. (2021). Elevated Pulmonary Pressure Noted on Echocardiogram: A Simplified Approach to Next Steps. J. Am. Heart Assoc..

[27] Tibshirani R. (2011). Regression shrinkage and selection via the lasso: A retrospective. J. R. Stat. Soc. Ser. B.

[28] Low A.H., Johnson S.R., Lee P. (2009). Ethnic influence on disease manifestations and autoantibodies in Chinese-descent patients with systemic sclerosis. J. Rheumatol..

[29] Al-Sheikh H., Ahmad Z., Johnson S.R. (2019). Ethnic Variations in Systemic Sclerosis Disease Manifestations, Internal Organ Involvement, and Mortality. J. Rheumatol..

[30] Morrisroe K., Stevens W., Sahhar J., Ngian G.S., Ferdowsi N., Hansen D., Patel S., Hill C.L., Roddy J., Walker J. (2020). The clinical and economic burden of systemic sclerosis related interstitial lung disease. Rheumatology.

[31] Jaeger V.K., Tikly M., Xu D., Siegert E., Hachulla E., Airo P., Valentini G., Matucci Cerinic M., Distler O., Cozzi F. (2020). Racial differences in systemic sclerosis disease presentation: A European Scleroderma Trials and Research group study. Rheumatology.

[32] Steen V., Domsic R.T., Lucas M., Fertig N., Medsger T.A. (2012). A clinical and serologic comparison of African American and Caucasian patients with systemic sclerosis. Arthritis Rheum..

[33] Mejia Otero C., Assassi S., Hudson M., Mayes M.D., Estrada Y.M.R., Pedroza C., Mills T.W., Walker J., Baron M., Stevens W. (2017). Antifibrillarin Antibodies Are Associated with Native North American Ethnicity and Poorer Survival in Systemic Sclerosis. J. Rheumatol..

[34] Hoa S., Baron M., Hudson M. (2021). Screening and management of subclinical interstitial lung disease in systemic sclerosis: An international survey. Rheumatology.

[35] Altman R.D., Medsger T.A., Bloch D.A., Michel B.A. (1991). Predictors of survival in systemic sclerosis (scleroderma). Arthritis Rheum..

[36] Domsic R.T., Nihtyanova S.I., Wisniewski S.R., Fine M.J., Lucas M., Kwoh C.K., Denton C.P., Medsger T.A. (2014). Derivation and validation of a prediction rule for two-year mortality in early diffuse cutaneous systemic sclerosis. Arthritis Rheumatol..

[37] Sehra S.T., Kelly A., Baker J.F., Derk C.T. (2016). Predictors of inpatient mortality in patients with systemic sclerosis: A case control study. Clin. Rheumatol..

[38] Wannarong T., Muangchan C. (2018). High burden of skin sclerosis is associated with severe organ involvement in patients with systemic sclerosis and systemic sclerosis overlap syndrome. Rheumatol. Int..

[39] Gayle A., Schoof N., Alves M., Clarke D., Raabe C., Das P., Del Galdo F., Maher T.M. (2020). Healthcare Resource Utilization Among Patients in England with Systemic Sclerosis-Associated Interstitial Lung Disease: A Retrospective Database Analysis. Adv. Ther..

[40] Moinzadeh P., Aberer E., Ahmadi-Simab K., Blank N., Distler J.H., Fierlbeck G., Genth E., Guenther C., Hein R., Henes J. (2015). Disease progression in systemic sclerosis-overlap syndrome is significantly different from limited and diffuse cutaneous systemic sclerosis. Ann. Rheum. Dis..

[41] Denton C.P. (2008). Renal manifestations of systemic sclerosis--clinical features and outcome assessment. Rheumatology.

[42] Ingraham K.M., O’Brien M.S., Shenin M., Derk C.T., Steen V.D. (2010). Gastric antral vascular ectasia in systemic sclerosis: Demographics and disease predictors. J. Rheumatol..

[43] Souma T., Yamazaki S., Moriguchi T., Suzuki N., Hirano I., Pan X., Minegishi N., Abe M., Kiyomoto H., Ito S. (2013). Plasticity of renal erythropoietin-producing cells governs fibrosis. J. Am. Soc. Nephrol. JASN.

[44] Kaneko K., Sato Y., Uchino E., Toriu N., Shigeta M., Kiyonari H., Endo S., Fukuma S., Yanagita M. (2022). Lineage tracing analysis defines erythropoietin-producing cells as a distinct subpopulation of resident fibroblasts with unique behaviors. Kidney Int..

[45] Valletta S., Thomas A., Meng Y., Ren X., Drissen R., Sengul H., Di Genua C., Nerlov C. (2020). Micro-environmental sensing by bone marrow stroma identifies IL-6 and TGFbeta1 as regulators of hematopoietic ageing. Nat. Commun..

[46] Weiss G., Ganz T., Goodnough L.T. (2019). Anemia of inflammation. Blood.

[47] Fang F., Marangoni R.G., Zhou X., Yang Y., Ye B., Shangguang A., Qin W., Wang W., Bhattacharyya S., Wei J. (2016). Toll-like Receptor 9 Signaling Is Augmented in Systemic Sclerosis and Elicits Transforming Growth Factor beta-Dependent Fibroblast Activation. Arthritis Rheumatol..

[48] Akilesh H.M., Buechler M.B., Duggan J.M., Hahn W.O., Matta B., Sun X., Gessay G., Whalen E., Mason M., Presnell S.R. (2019). Chronic TLR7 and TLR9 signaling drives anemia via differentiation of specialized hemophagocytes. Science.

[49] Mitev A., Christ L., Feldmann D., Binder M., Moller K., Kanne A.M., Hugle T., Villiger P.M., Voll R.E., Finzel S. (2019). Inflammatory stays inflammatory: A subgroup of systemic sclerosis characterized by high morbidity and inflammatory resistance to cyclophosphamide. Arthritis Res. Ther..

[50] Hamaguchi Y., Kodera M., Matsushita T., Hasegawa M., Inaba Y., Usuda T., Kuwana M., Takehara K., Fujimoto M. (2015). Clinical and immunologic predictors of scleroderma renal crisis in Japanese systemic sclerosis patients with anti-RNA polymerase III autoantibodies. Arthritis Rheumatol..

[51] Motegi S., Toki S., Yamada K., Uchiyama A., Ishikawa O. (2015). Demographic and clinical features of systemic sclerosis patients with anti-RNA polymerase III antibodies. J. Dermatol..

[52] Liu C., Hou Y., Xu D., Li L., Zhang Y., Cheng L., Yan S., Zhang F., Li Y. (2020). Analysis of anti-RNA polymerase III antibodies in Chinese Han systemic sclerosis patients. Clin. Rheumatol..

