# Supplementary material for: Anemia Is an Indicator for Worse Organ Damage Trajectories in Patients with Systemic Sclerosis: A Retrospective Study
Source: J Clin Med. 2022 Aug 26;11(17):5013. doi: 10.3390/jcm11175013 (PMC9456668; doi:10.3390/jcm11175013)
Supplement: Supplementary file 1 [file jcm-11-05013-s001.zip › jcm-1833008-supplementary.pdf]

Supplementary files:

Table S1. Frequency of SCTC-DI items in different SSc cohorts

Items	PKUTH and PKUPH-SSc N = 433	ASCS N = 1568	*p* value	CSRG N = 1624	*p* value
Musculoskeletal and skin					
Joint contracture (small joints)	56 (12.9%)	827 (52.7%)	< 0.001	584 (36.0%)	< 0.001
Joint contracture (large joints)	7 (1.6%)	536 (34.2%)	< 0.001	305 (18.8%)	< 0.001
Sicca symptoms	160 (37.0%)	806 (51.4%)	< 0.001	785 (48.3%)	< 0.001
Proximal muscle weakness	32 (7.4%)	72 (4.6%)	0.020	236 (14.5%)	< 0.001
Calcinosis complicated by infection or requiring surgery	7 (1.6%)	46 (3.0%)	0.131	-	-
Vascular					
Digital ulceration	87 (20.1%)	440 (28.1%)	0.001	164 (16.3%)	< 0.001
Digital amputation required	19 (4.4%)	149 (9.5%)	0.001	134 (8.3%)	0.006
Gastrointestinal					
Esophageal dysmotility	69 (15.9%)	143 (9.1%)	< 0.001	1160 (71.4%)	< 0.001
Esophageal stricture	2 (0.5%)	171 (10.9%)	< 0.001	218 (13.4%)	< 0.001
Refractory gastro-esophageal reflux disease (heartburn)	103 (23.8%)	747 (47.6%)	< 0.001	1029 (63.4%)	< 0.001
GAVE	0 (0.0%)	115 (7.3%)	-	-	-
Pseudo-obstruction	6 (1.4%)	45 (2.9%)	0.083	146 (9.0%)	< 0.001
BMI < 18.5 kg/m^2 or weight loss > 10% in the last 12 months	155 (35.8%)	801 (51.1%)	< 0.001	361 (22.2%)	< 0.001
Respiratory					
ILD > 20% extent on HRCT	172 (39.7%)	141 (9.0%)	< 0.001	365 (22.5%)	< 0.001
FVC < 70%	52 (12.0%)	60 (3.8%)	< 0.001	39 (7.1%)	< 0.001
Dependence on home oxygen	9 (2.1%)	59 (3.8%)	0.087	117 (7.2%)	< 0.001
Cardiovascular					
PAH	42 (9.7%)	249 (15.9%)	0.001	125 (7.7%)	0.175
Moderate to severe right ventricular dysfunction	10 (2.3%)	264 (16.8%)	< 0.001	-	-
Myocardial disease	22 (5.1%)	107 (6.8%)	0.191	81 (5.0%)	0.937
Moderate to large pericardial effusion	13 (3.0%)	-	-	45 (2.8%)	0.796
Renal					
History of SRC	5 (1.2%)	39 (2.5%)	0.094	77 (4.7%)	0.001
eGFR < 45 ml/min/1.73m^2	4 (0.9%)	7 (0.5%)	0.411	34 (2.1%)	0.160
CKD stage 5 and need for renal replacement therapy	1 (0.2%)	4 (0.3%)	1.000	33 (2.0%)	0.016

Abbreviations: SCTC-DI, Scleroderma Clinical Trials Consortium Damage Index; SSc: systemic sclerosis; PKUTH-SSc, Peking University Third Hospital SSc cohort; PKUPH-SSc, Peking University People's Hospital SSc cohort; ASCS, Australian Scleroderma Cohort Study cohort; CSRG, Canadian Scleroderma Research Group cohort; GAVE, Gastric antral vascular ectasia; BMI, body mass index; ILD, interstitial lung disease; HRCT, HRCT, high-resolution CT; FVC, Forced Vital Capacity; PAH, pulmonary arterial hypertension; SRC, scleroderma renal crisis; eGFR, Estimated Glomerular Filtration Rate; CKD, chronic kidney disease.
Note: *p* value represents the frequency differences compared between ASCS cohort and our cohort, CSRG cohort and our cohort, respectively;

Data of ASCS and CSRG cohorts were adapted from "Development and validation of the Scleroderma Clinical Trials Consortium Damage Index (SCTC-DI): a novel instrument to quantify organ damage in systemic sclerosis," by Ferdowsi N, *et al.*, 2019, *Ann Rheum Dis, 78*(6), Supplementary File. Copyright statement. Missing data of ASCS and CSRG cohorts have been excluded.

Table S2. Clinical characteristics at baseline of SCTC-DI low-burden (< 6) and high-burden (≥ 6) group

Characteristics	Low-burden N = 273	High-burden N = 160	*p* value
Age at initial visit (years)	49.8 ± 15.3	55.8 ± 12.0	< 0.001
Sex, female	229 (83.9%)	144 (90.0%)	0.075
Disease duration (years)	7.1 ± 9.0	10.0 ± 10.1	0.003
Disease classification			
lcSSc	103 (37.7%)	70 (43.8%)	0.217
dcSSc	118 (43.2%)	59 (36.9%)	0.195
Sine scleroderma	10 (3.7%)	3 (1.9%)	0.447
Overlap syndrome	42 (15.4%)	28 (17.5%)	0.564
RA	21 (7.7%)	6 (3.8%)	0.102
SLE	17 (6.2%)	12 (7.5%)	0.609
DM/PM	4 (1.5%)	10 (6.3%)	0.007
Laboratory parameters			
Anemia	86 (31.5%)	89 (55.6%)	< 0.001
High ESR	83/255 (32.5%)	73/154 (47.4%)	0.003
High CRP	50/253 (20.6%)	35/150 (23.3%)	0.519
Hypocomplementemia	102/174 (42.5%)	74/79 (49.0%)	0.208
Autoantibody profile			
ANA	193 (70.7%)	119 (74.4%)	0.410
Anti-topoisomerase 1 (Anti-Scl-70)	75 (27.5%)	54 (33.8%)	0.168
Anti-centromere proteins	40 (14.7%)	16 (10.0%)	0.164
Medication			
Steroids	108 (39.6%)	83 (51.9%)	0.013
Immunosuppressants	103 (37.7%)	62 (38.8%)	0.833

N = 273 in anemia group and N = 160 in non-anemia group if not specified.

Abbreviations: SCTC-DI, Scleroderma Clinical Trials Consortium Damage Index; lcSSc, limited cutaneous systemic sclerosis; dcSSc, diffused cutaneous systemic sclerosis; RA, rheumatoid arthritis; SLE, systemic lupus erythematous; DM, dermatomyositis; PM, polymyositis; ESR, erythrocyte sedimentation rate; CRP: C-reactive protein; ANA, anti-nuclear antibody.

Table S3. Clinical characteristics of SCTC-DI modified low-burden (< 4) and high-burden (≥ 4) group

Characteristics	Low-burden N = 175	High-burden N = 258	*p* value
Age at initial visit (years)	49.9 ± 15.4	53.5 ± 13.6	0.012
Sex, female	143 (81.7%)	230 (89.1%)	0.028
Disease duration (years)	5.7 ± 8.5	9.9 ± 9.8	<0.001
Disease classification			
lcSSc	69 (39.4%)	104 (40.3%)	0.854
dcSSc	75 (42.9%)	102 (39.5%)	0.490
Sine scleroderma	5 (2.9%)	8 (3.1%)	0.884
Overlap syndrome	26 (14.9%)	44 (17.1%)	0.542
RA	16 (9.1%)	11 (4.3%)	0.039
SLE	8 (4.6%)	21 (8.1%)	0.145
DM/PM	2 (1.1%)	12 (4.7%)	0.043
Laboratory parameters			
Anemia	52 (29.7%)	123 (47.7%)	< 0.001
High ESR	50/166 (30.1%)	106/243 (43.6%)	0.006
High CRP	30/154 (19.5%)	55/239 (23.0%)	0.406
Hypocomplementemia	69/156 (44.2%)	107/235 (45.5%)	0.800
Autoantibody profile			
ANA	121 (69.1%)	191 (74.0%)	0.266
Anti-topoisomerase 1 (Anti-Scl-70)	44 (25.1%)	85 (32.9%)	0.081
Anti-centromere proteins	23 (13.1%)	33 (12.8%)	0.915
Medication			
Steroids	69 (39.4%)	122 (47.3%)	0.106
Immunosuppressants	66 (37.7%)	99 (38.4%)	0.890

N = 175 in low-burden group and N = 258 in high-burden group if not specified.

Abbreviations: SCTC-DI, Scleroderma Clinical Trials Consortium Damage Index; lcSSc, limited cutaneous systemic sclerosis; dcSSc, diffused cutaneous systemic sclerosis; RA, rheumatoid arthritis; SLE, systemic lupus erythematous; DM, dermatomyositis; PM, polymyositis; ESR, erythrocyte sedimentation rate; CRP: C-reactive protein; ANA, anti-nuclear antibody.

Table S4. Frequency of SCTC-DI items in SSc patients during follow-up (N = 207)

Items	Baseline assessment	Follow-up assessment	Progression
Musculoskeletal and skin	106 (51.2%), Score: 1.78 ± 2.03	125 (60.4%), Score: 1.03 ± 1.99	22 (10.6%)
Joint contracture (small joints)	26 (12.6%)	35 (16.9%)	9 (4.3%)
Joint contracture (large joints)	2 (1.0%)	3 (1.4%)	1 (0.4%)
Sicca symptoms	87 (42.0%)	95 (45.9%)	8 (3.8%)
Proximal muscle weakness	13 (6.3%)	16 (7.7%)	3 (1.4%)
Calcinosis complicated by infection or requiring surgery	3 (1.4%)	3 (1.4%)	0 (0.0%)
Vascular	36 (17.4%), Score: 0.36 ± 0.79	46 (22.2%), Score: 0.46 ± 0.88	11 (5.3%)
Digital ulceration	36 (17.4%)	46 (22.2%)	10 (4.8%)
Digital amputation required	2 (1.0%)	4 (1.9%)	2 (0.9%)
Gastrointestinal	100 (48.3%), Score: 0.99 ± 1.22	126 (60.9%), Score: 1.28 ± 1.33	41 (19.8%)
Esophageal dysmotility	32 (15.5%)	38 (18.4%)	6 (2.9%)
Esophageal stricture	2 (1.0%)	2 (1.0%)	0 (0.0%)
Refractory gastro-esophageal reflux disease (heartburn)	45 (21.7%)	68 (32.9%)	23 (11.1%)
GAVE	0 (0.0%)	0 (0.0%)	0 (0.0%)
Pseudo-obstruction	1 (0.5%)	2 (1.0%)	1 (0.4%)
BMI < 18.5 kg/m^2 or weight loss > 10% in the last 12 months	61 (29.5%)	75 (36.2%)	14 (6.7%)
Respiratory	86 (41.5%), Score: 1.34 ± 2.17	117 (56.5%), Score: 1.97 ± 2.53	39 (18.8%)
ILD > 20% extent on HRCT	84 (40.6%)	117 (56.5%)	33 (15.9%)
FVC < 70%	21 (10.1%)	35 (16.9%)	14 (6.7%)
Dependence on home oxygen	5 (2.4%)	7 (3.4%)	2 (0.9%)
Cardiovascular	19 (9.2%), Score: 0.25 ± 0.98	28 (13.5%), Score: 0.44 ± 1.37	14 (6.8%)
PAH	13 (6.3%)	23 (11.1%)	10 (4.8%)
Moderate to severe right ventricular dysfunction	1 (0.5%)	2 (1.0%)	1 (0.4%)
Myocardial disease	5 (2.4%)	10 (4.8%)	5 (2.4%)
Moderate to large pericardial effusion	5 (2.4%)	5 (2.4%)	0 (0.0%)
Renal	0 (0.0%), Score: 0.00 ± 0.00	3 (1.4%), Score: 0.06 ± 0.54	3 (1.4%)
History of SRC	0 (0.0%)	3 (1.4%)	3 (1.4%)
eGFR < 45 ml/min/1.73m^2	0 (0.0%)	2 (1.0%)	2 (0.9%)
CKD stage 5 and need for renal replacement therapy	0 (0.0%)	1 (0.5%)	1 (0.4%)
SCTC-DI	4.71 ± 3.98	6.24 ± 4.46	/
SCTC-DI = 0	34 (16.4%)	13 (6.3%)	/
ΔSCTC-DI ≥ 1 (Follow-up)	/	/	100 (48.3%)

Abbreviations: SCTC-DI, Scleroderma Clinical Trials Consortium Damage Index; SSc: systemic sclerosis; GAVE, Gastric antral vascular ectasia; BMI, body mass index; ILD, interstitial lung disease; HRCT, HRCT, high-resolution CT; FVC, Forced Vital Capacity; PAH, pulmonary arterial hypertension; SRC, scleroderma renal crisis; eGFR, Estimated Glomerular Filtration Rate; CKD, chronic kidney disease.

Table S5. Univariate Cox regression analysis of the organ damage progression based on SCTC-DI (N = 207)

Baseline Characteristics	Univariate analysis		Multivariate analysis	
	HR (95% CI)	*p* value	HR (95% CI)	*p* value
Age ≥ 60 years	0.87 (0.55, 1.35)	0.524		
Sex (female)	0.86 (0.48, 1.54)	0.612		
Duration ≥ 2 years	0.77 (0.52, 1.16)	0.209		
Classification				
lcSSc	0.68 (0.43, 1.07)	0.094		
dcSSc	1.11 (0.75, 1.65)	0.615		
Sine scleroderma	0.93 (0.13, 6.65)	0.939		
Overlap	1.34 (0.87, 2.08)	0.189		
RA	1.47 (0.82, 2.63)	0.199		
SLE	1.27 (0.70, 2.33)	0.435		
DM/PM	0.84 (0.27, 2.65)	0.765		
Anemia	1.59 (1.07, 2.36)	0.022	1.75 (1.16, 2.66)	0.008
High ESR (n = 192)	1.54 (1.03, 2.31)	0.036		
High CRP (n = 190)	1.74 (1.12, 2.69)	0.014		
Hypocomplementemia (n= 190)	1.33 (0.88, 2.00)	0.175		
Autoantibody profile				
ANA	1.03 (0.66, 1.62)	0.894		
Anti-Scl70	1.53 (1.00, 2.34)	0.048	1.91 (1.22, 2.99)	0.005
Anti-centromere proteins	0.61 (0.31, 1.17)	0.133		
Medicine usage				
Initial steroids	1.09 (0.73, 1.63)	0.687		
Initial Immunosuppressants	0.77 (0.50, 1.18)	0.232		
Follow-up steroids	1.72 (0.99, 2.99)	0.053		
Follow-up Immunosuppressants	0.95 (0.63, 1.44)	0.816		
SCTC-DI damage burden at initial				
High burden (≥ 6)	0.80 (0.51, 1.25)	0.325		
Modified high burden (≥ 4)	0.82 (0.55, 1.21)	0.311		

N = 207 if not specified.

Abbreviations: SCTC-DI, Scleroderma Clinical Trials Consortium Damage Index; lcSSc, limited cutaneous systemic sclerosis; dcSSc, diffused cutaneous systemic sclerosis; RA, rheumatoid arthritis; SLE, systemic lupus erythematous; DM, dermatomyositis; PM, polymyositis; ESR, erythrocyte sedimentation rate; CRP: C-reactive protein; ANA, anti-nuclear antibody.

Table S6. Organ damage incident in different organ systems in SSc patients with and without anemia.

Organ systems	Anemia N=175	Non-anemia N=258	*p* value
Musculoskeletal and skin	106 (60.6%)	102 (39.5%)	< 0.001
Vascular	36 (20.6%)	51 (19.8%)	0.838
Gastrointestinal	109 (62.3%)	115 (44.6%)	< 0.001
Respiratory	76 (43.4%)	100 (38.8%)	0.332
Cardiovascular	29 (16.6%)	26 (10.1%)	0.046
Renal	6 (3.4%)	0 (0.0%)	0.010
SCTC-DI = 0	17 (9.7%)	51 (19.8%)	0.005

Abbreviations: SSc, systemic sclerosis; SCTC-DI, Scleroderma Clinical Trials Consortium Damage Index.

Table S7. Organ damage progression incident in different organ systems in SSc patients with and without anemia

**Organ Systems**	**Anemia N=83**	**Non-anemia N=124**	***p* value**
Musculoskeletal and skin	7 (8.4%)	15 (12.1%)	0.402
Vascular	3 (3.6%)	8 (6.5%)	0.565
Gastrointestinal	21 (25.3%)	20 (16.1%)	0.105
Respiratory	20 (24.1%)	19 (15.3%)	0.114
Cardiovascular	10 (12.0%)	4 (3.2%)	0.013
Renal	3 (3.6%)	0 (0.0%)	0.033
ΔSCTC-DI ≥ 1	50 (60.2%)	50 (40.3%)	0.005

Abbreviations: SSc, systemic sclerosis; SCTC-DI, Scleroderma Clinical Trials Consortium Damage Index.

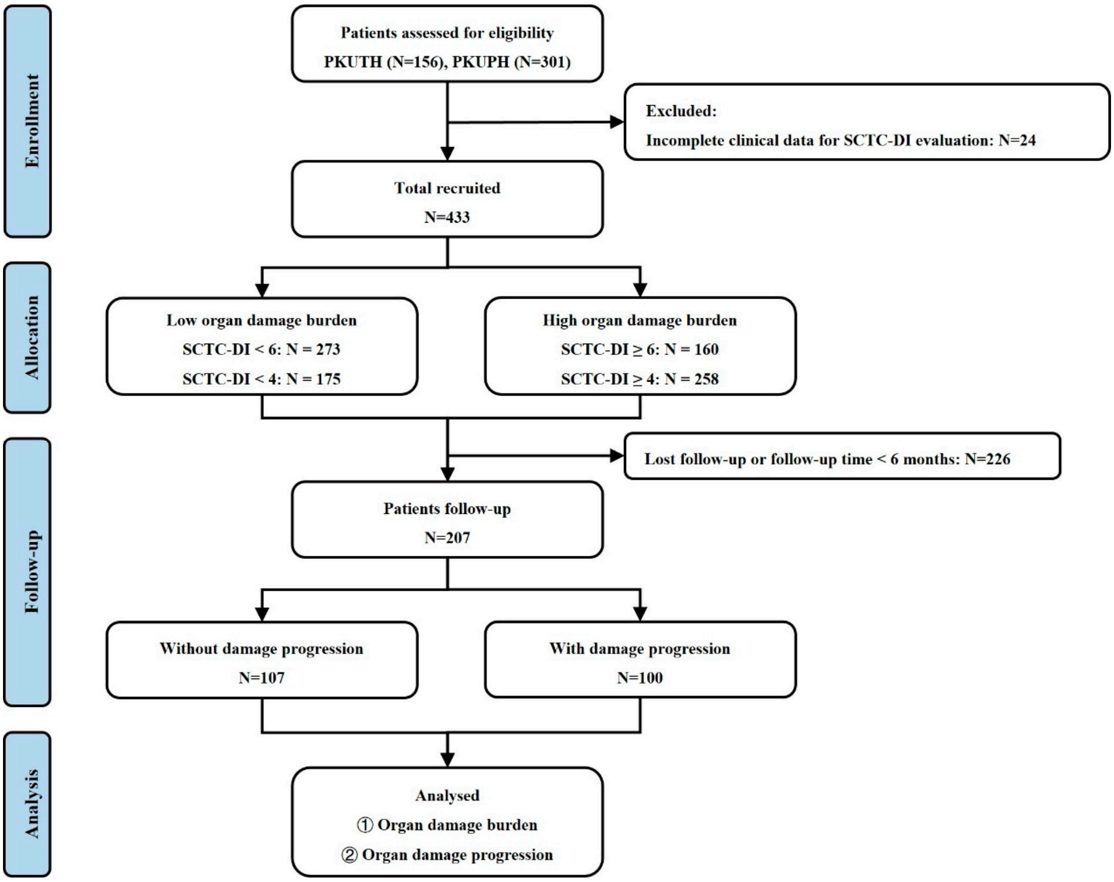

Figure S1. Study diagram. A simple study flow diagram of our study.
